# Supplementary material for: Screening the Pathogen Box to Discover and Characterize New Cruzain and TbrCatL Inhibitors
Source: Pathogens. 2023 Feb 4;12(2):251. doi: 10.3390/pathogens12020251 (PMC9967275; doi:10.3390/pathogens12020251)
Supplement: Supplementary file 1 [file pathogens-12-00251-s001.zip › SupplementaryMaterial2.pdf]

Supplementary Material

Screening the Pathogen box to discover and characterize new cruzain and *Tbr*CatL inhibitors

Thales do Valle Moreira [1]†, Luan Carvalho Martins [1]†, Lucas Abreu Diniz [1], Talita Cristina Diniz Bernardes [1], Renata Barbosa de Oliveira [2], and Rafaela Salgado Ferreira [1,*]

[1] Molecular Modeling and Drug Design Laboratory. Institute for Biological Sciences. Federal University of Minas Gerais. 6627, Antônio Carlos Avenue. 31270-901. Belo Horizonte-MG, Brazil.
[2] Pharmaceutical Products Department. Federal University of Minas Gerais. 6627, Antônio Carlos Avenue. 31270-901. Belo Horizonte-MG, Brazil.
* Correspondence: rafaelasf@icb.ufmg.br
† these authors contributed equally to this work

Table S1 – Effect of **MMV676409** and **MMV676512** in the inicial velocitities of the enzymatic reactions, after 10 minutes of pre-incubation of the enzyme and the compound. All effects were calculated in respect to the DMSO control.

**Compound**	**Increase in the initial velocity of the reaction (%)**					
	**5 µM using PB samples[a]**		**100 µM using solid samples[b]**		**5 µM using salid samples[b]**	
	**Cruzain**	***Tbr*CatL**	**Cruzain**	***Tbr*CatL**	**Cruzain**	***Tbr*CatL**
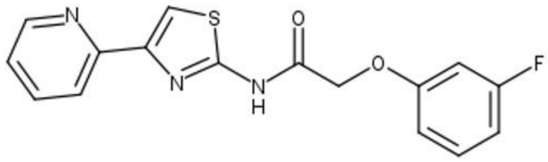 **MMV676409**	510 ± 216	772 ± 382	0 ± 6	*ND*	-4 ± 2	*ND*
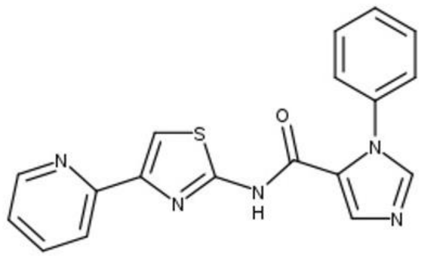 **MMV676512**	84 ± 54	109 ± 71	24 ± 5	*ND*	14 ± 9	*ND*

[a]: experiments performed using the solutions from the PB compound library. [b]: experiments performed using solutions prepared from the solid samples. *ND*: not determined.

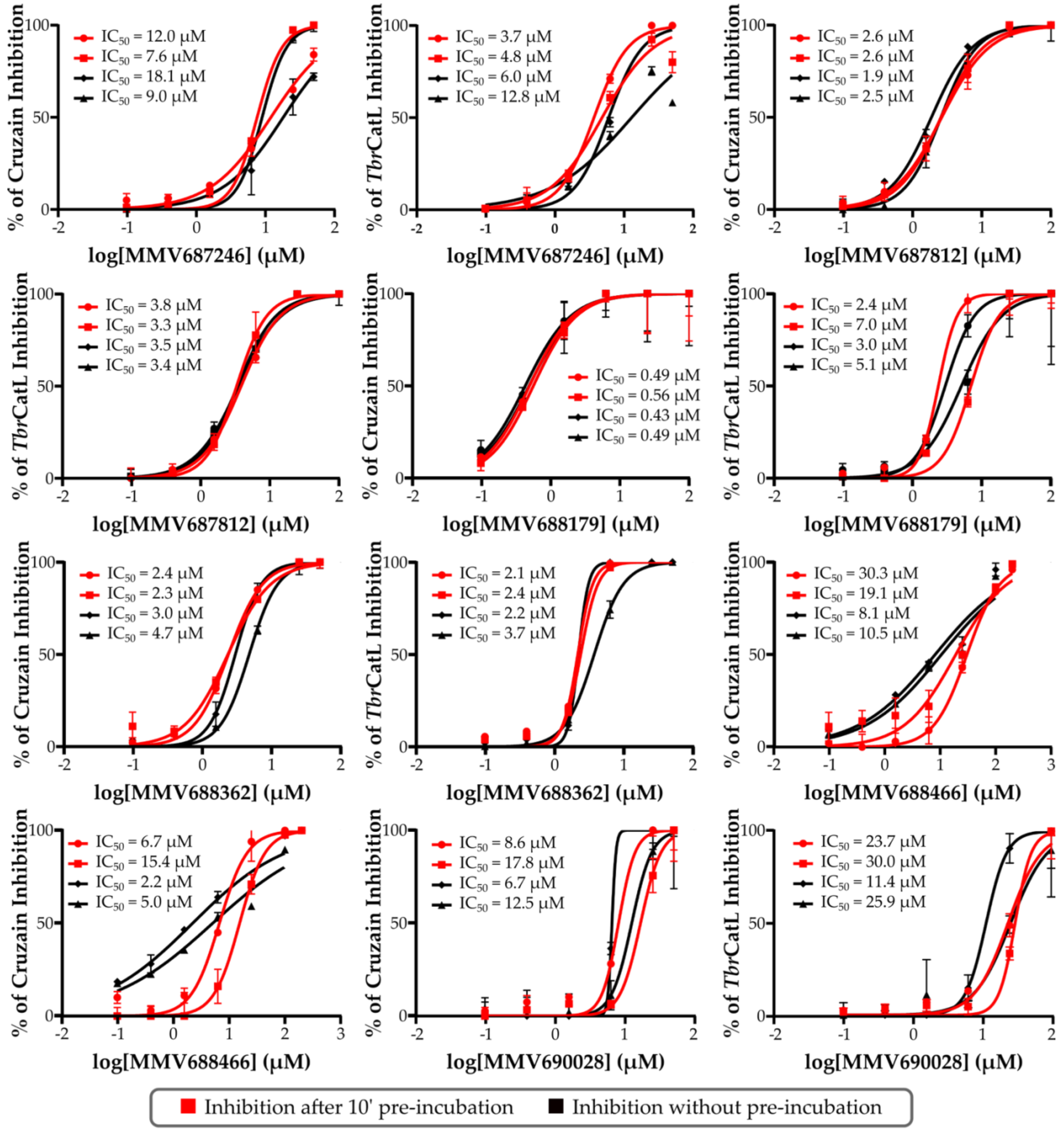

Figure S1 – IC_{50} curves for compounds **MMV687246**, **MMV687812**, **MMV688179**, **MMV688362**, **MMV688466**, **MMV690028** against cruzain and *Tbr*CatL. For each compound/enzyme pair, four curves are reported, two obtained in experiments with a ten minute pre-incubation of the compounds with the enzymes (red) and two obtained in experiments without pre-incubation (black curves). Each curve was determined based on at least seven compound concentrations, in triplicate. All percentages of inhibition were calculated based on a DMSO control.

Table S2 – Cruzain inhibition by hits from the initial screening, at varying assay conditions.

Compound	Concentration of the compound evaluated (μM)	% Cruzain Inhibition (Mean ± SEM) [a]			
		0.1% Triton X-100	0.01% Triton X-100	0.001% Triton X-100	0.001% Triton + BSA 1 mg/mL
**MMV687246**	12.5	32 ± 2	51 ± 0	62 ± 3	45 ± 1
**MMV687812**	3.125	54 ± 1	95 ± 0	92 ± 0	85 ± 1
**MMV688179**	1.563	82 ± 13	80 ± 1	85 ± 6	89 ± 8
**MMV688362**	3.125	25 ± 2	41 ± 2	59 ± 2	29 ± 3
**MMV688466**	6.25	82 ± 11	32 ± 5	83 ± 3	96 ± 3
**MMV690028**	25	100 ± 0	100 ± 0	69 ± 14	91 ± 7

[a]: mean over two independent experiments, in triplicates.

Table S3 – Cruzain inhibition by analogues of compound **MMV688179.**

**Compound**	**Structure** 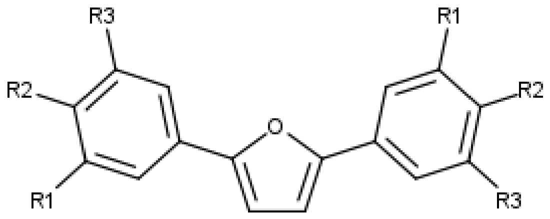 **R1**	**R2**	**R3**	**% Cruzain Inhibition at 100 µM (Mean ± SEM)** [a]	**Comments**
**1**	H	H	H	ND	Solubility issues
**2**	H	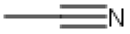	H	0 ± 6	-
**3**	H	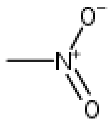	H	ND	Solubility issues
**4**	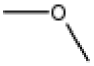	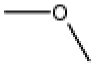	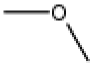	ND	Fluorescence, interference with the assay readout
**5**	H	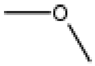	H	ND	Fluorescence, interference with the assay readout
**6**	H	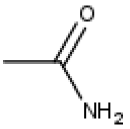	H	57 ± 4	Fluorescence, interference with the assay readout
**7**	H	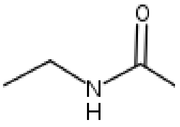	H	65 ± 4	Insoluble at concentrations higher than 100 µM

ND = not determined [a]: mean over two independent experiments, in triplicates.
